# Transfer of Ethanol and Aroma Compounds by Varying Specific Process Parameters in the Thermal Dealcoholisation of Beer

**DOI:** 10.3390/foods10071602

**Published:** 2021-07-10

**Authors:** Magdalena Müller, Thomas Becker, Martina Gastl

**Affiliations:** Research Group Raw Material Based Brewing and Beverage Technology, Institute of Brewing and Beverage Technology, Technical University of Munich, 85354 Freising, Germany; magdalena.mueller@tum.de (M.M.); tb@tum.de (T.B.)

**Keywords:** non-alcoholic beer, dealcoholisation, aroma components, water-ethanol mixtures, recovery, thermal separation

## Abstract

Dealcoholisation of beer has gained prominence over the last decade. A well-known procedure involves the combination of a rectification column for thermal dealcoholisation and a downstream column for aroma recovery. However, the recovery of valuable fermentation by-products is rarely performed due to limited data about the enrichment and depletion of ethanol and aromatic compounds. The influence of operating conditions on the transfer of ethanol and aroma compounds to the recovery fluid, henceforth, ‘aromawater’, has not yet been fully explored. Therefore, this study involved examining how ethanol concentration and aroma compounds in the aromawater are affected by the condenser temperature and reflux rate during thermal dealcoholisation. The aim was to obtain an aromawater having a maximum level of valuable aroma substances and a minimum level of ethanol for re-blending with non-alcoholic beer, hypothetically causing aroma intensification. An industrial system was used for sample production. Ethanol as well as higher alcohols and ester concentrations were analysed in the different material flows, and mass balances were thus compiled. Sensory analysis was performed to evaluate the beer aroma’s intensification as a sustainable industrial application. The obtained results indicate that increased condenser temperature was associated with increased aroma concentrations in the aromawater. If the temperature of the condenser’s coolant exceeded 15 °C, dealcoholisation < 0.05% abv could not be guaranteed. A higher reflux rate led to higher concentrations of fermentation by-products in the aromawater. Finally, the aroma profile of three non-alcoholic beers (0.0% abv, 0.5% abv after blending with original beer, and 0.5% abv after blending with aromawater) were evaluated. By blending, the attributes ‘estery’ and ‘flowery’ were assessed as dominant. The effect was more pronounced with aromawater than with the original beer.

## 1. Introduction

In 2020, the global market value for non-alcoholic beer (NAB) was estimated at around USD 18 billion. For years to come, the same global market is expected to grow at an annual rate of 7.5% and reach an estimated value of USD 25 billion by 2024 [1]. In Germany, for example, the production volume of non-alcoholic beer has almost doubled over the last ten years, from 230 million litres (2009) to 420 million litres (2019) [2]. Moreover, worldwide, NABs and low-alcohol beers are often referred to as the ‘trend of the decade’. These products’ beneficial nutritional value and health effects, in combination with increased health awareness among the general public as well as among policy makers, have spurred this development [3,4]. Furthermore, the marketing of breweries is possible without ethical, moral or legal drawbacks, unlike advertising products containing harmful ingredients.

From an engineering point of view, dealcoholisation is a separation process. Besides membrane dealcoholisation, thermal processes are a widely employed method for producing NABs, and can be realised via different procedures (e.g., evaporation, rectification and spinning cone column). The objective of these processes is to separate ethanol (EtOH) from alcoholic matrices, which are homogenous mixtures (e.g., wine, cider or as in this study, beer), as selectively as possible by exploiting differences in boiling points and evaporation characteristics [5].

The surface of the rectification column used in this study increased by featuring structured, β-fold sheets of metal packages. Owing to this additional surface, the components of the water–EtOH mixture containing highly dilute aroma compounds (i.e., the original beer, *beer OB*) are separated according to its vapour–liquid equilibrium (VLE). As a part of the process, beer OB is to be separated via successive distillation steps into *NAB* (the aqueous phase, low-boiling-point components) and condensate (*CON*; the vapour phase, high-boiling-point components, including EtOH and aroma compounds). The volatilities of valuable fermentation by-products like aliphatic alcohols (e.g., 2-methylpropanol, 2-methylbutanol and 3-methylbutanol) and the ester (3-methylbutyl acetate) are similar to that of EtOH; therefore, the mentioned chemicals are lost during the dealcoholisation process.

In the system, a second rectification column is placed after the dealcoholisation unit; this column is affiliated to the condenser, and its purpose is to recover aroma compounds from the high-boiling fraction. In this column, degassed water represents the low-boiling fraction, whereas the not-fluidised substances in the condenser represent the high-boiling one. The fluid obtained as a part of the aroma recovery procedure is hereafter referred to as ‘aromawater’ (*AW*).

The thermal load on the product to be dealcoholised is often discussed as a deficit of thermal dealcoholisation methods [6]. The VLE is determined by temperature and pressure. The thermal dealcoholisation process is performed under vacuum to decrease the saturation vapour pressure of the individual components and thus decrease the thermal load on the product. As products of thermal processes in brewing, the evaporation and transformation of aroma and bittering compounds in the brewhouse (dimethyl sulphide and humulone) are discussed [7,8,9]. Moreover, the loss of aroma compounds such as ethyl acetate and 3-methylbutyl acetate is known to result from stripping during fermentation [10]. However, knowledge acquired from these studies can be applied to the description of thermal dealcoholisation only to a limited extent. In brewing technology, physico-chemical analyses (e.g., for the residual extract, colour, pH, bittering units and thiobarbituric acid index) and sensory evaluation are employed to define the quality of a NAB. The results of dealcoholisation experiments conducted with a falling film evaporator [11] and a counter-current distillation system [12] indicated that NAB’s quality-relevant characteristics and flavour could be influenced by varying the evaporation temperature and the degree of dealcoholisation (resultant EtOH concentration 0.12–0.66% abv). Although subsequent steps required in order to improve the resulting NAB’s aroma profile by re-blending the aroma fraction have previously been evaluated [11,12], techniques for influencing the composition of the dealcoholisation by-product with respect to its EtOH and aroma compound concentration remain unknown.

The lower the final EtOH concentration in the NAB, the higher the loss of aroma substances [12]. The possibilities for aroma and sensory enhancement after beer dealcoholisation in Germany are dictated by the Purity Law, and they are mostly associated with a slight increase of the NAB’s EtOH level, and thus limited to use in the production of 0.0% abv NABs. Nevertheless, this addition of original beer aroma compounds is a tool that can be employed to intensify the typical beer flavours, especially in the case of estery top-fermented NABs [12]. For this reason, top-fermented wheat beers were used in this study.

Breweries that are not committed to the German Purity Law are allowed to add flavouring substances to their product. The question in this case is which compounds should be added and in what ratios, to achieve a type-typical beer flavour, because thresholds and concentration-effect relationships are different between NABs and alcoholic beers [13,14,15,16,17]. Aroma recovery of beer flavourings is also possible by implementing membrane processes such as pervaporation [18,19]. However, to the best of our knowledge, no industrial application of this membrane distillation procedure has been performed on a large scale.

Considering sustainability, the enrichment of valuable aroma components in the AW is desirable, as AW can be reused, in contrast to the CON. Therefore, it is important to identify the aroma compounds or fractions that are enriched in the AW and correlations with the EtOH levels in the CON.

Investigating the recovery of the aroma substances formed during the brewing process, its shift and transfer mechanisms into the rectification process’s mass flows CON and AW is the objective of this study. Therefore, the effect of varying the values of the two parameters condenser temperature (T_CON_) and reflux rate ($\dot{V}$_REFLUX_) was explored. The aim of this study was to obtain AW characterised by high concentrations of fermentation by-products and a low EtOH concentration, such that it can be used to improve the sensory evaluation scores of NABs.

The authors used an industry-standard unit for tracking the EtOH and fermentation by-products in the different mass flows. The authors intend to show how the type and quality of recovered flavourings can be influenced by the choice of the values of parameters T_CON_ and $\dot{V}$_REFLUX_. In a second step of the investigation as a practical application of the study findings, we evaluated whether aroma optimisation of NABs can be achieved by blending the dealcoholisation by-product AW.

## 2. Materials and Methods

### 2.1. Thermal Dealcoholisation Process Setup

**Figure 1:**
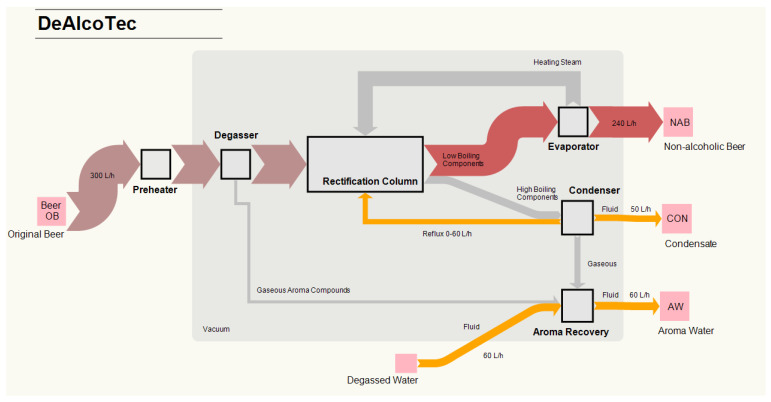
Representation of the flow scenarios in the DeAlcoTec^®^ thermal dealcoholisation system, which was used to process the original beer (beer OB) samples. The pink squares represent the initial beer OB and final products non-alcoholic beer (NAB), condensate (CON) and aromawater (AW). The quantitative flows of the individual gaseous (grey arrows) and liquid (red, orange and lilac pale blue arrows) media illustrate the continuous dealcoholisation and aroma recovery processes, which are linked as an overall process. The black square rectangles identify the single unit operations in the system.

The industrial-scale system DeAlcoTec^®^ (Centec GmbH, Maintal, Germany) was used for NAB production. The system’s design is schematically depicted in Figure 1.

Before the actual dealcoholisation, the beer passes through a preheater (5 °C to 25 °C), designed as a regenerative zone, and through a degasser, where CO_2_ is separated and forwarded to the aroma recovery section. The system is equipped with a vacuum pump to reduce the thermal load. The degasser, rectification column, falling film evaporator, condenser and aroma recovery are connected; thus, the pressure is identical in these systems’ components (40–100 mbar).

Under conditions of intensive exchange of mass and heat, the separation and dealcoholisation take place in the rectification column. The subsequent evaporator gains the vapour phase for performing the counter-current distillation. In the column, beer represents the low-boiling fraction; the high-boiling components (i.e., EtOH and aroma compounds) are passed over to the condenser. Depending on the temperature in the condenser, a new VLE sets. The fluid CON can be forwarded to the rectification column as reflux, which leads to a further increase of the EtOH in the CON, or it can be discharged from the process as an intermediate product. The volatile parts that are not liquefied in the condenser are transferred to the aroma recovery section, where they are brought into contact with degassed water (see Figure 1). Tracking the aroma transfer within the system is not possible due to the vacuum environment. The adopted tracking setup involved installing a three-way valve to sample CON.

For each parameter set of the dealcoholisation, the relevant experiment was conducted in triplicate. The volume flow rate of beer OB was 300 L h^−1^, and a vacuum of 40 mbar was applied. The chosen evaporation temperature T_EVAP_ was 36 °C. The value of T_CON_ was varied from 10 °C to 25 °C and the $\dot{V}$_REFLUX_ value from 0 to 60 L h^−1^. The flow rate of the degassed water into the aroma recovery ($\dot{V}$_AroRec_) was 60 L h^−1^. The temperature of the aroma recovery remained constant at 8 °C.

To quantise the degree of recovery in the AW, the factor c_REL_ was introduced. This parameter provides information on the proportion of the EtOH or flavouring substance that is transferred from the CON into the AW. c_REL_ thus represents the percentage of aroma substance concentration in the AW divided by its level in the CON.

c_REL_ = [c(AW) × 100]/c(CON) (1)

### 2.2. Sample Material

The products to be subjected to the dealcoholisation process were brewed in the Research Brewery Weihenstephan according to standard recipe for top-fermented wheat beers. Fermentation was conducted with the yeast strain SafAle US 05 (Fermentis by Lesaffre, Paris, France). The level of EtOH was 5.28 ± 0.07% abv. The original gravity was 12.14 ± 0.15% mas, the residual extract was 4.16 ± 0.07% mas, the apparent extract was 2.25 ± 0.05% mas and the pH was 4.47 ± 0.15.

The samples to be subjected to sensory evaluation were blended after dealcoholisation. The amount of beer and AW was calculated based on the EtOH content of the media. The target alcohol level was 0.5% abv, as this is the legal limit for NABs in the US, UK and Germany [20].

### 2.3. EtOH and Aroma Compound Quantification

The amounts of EtOH and aroma compounds were measured according to approved standard Mitteleuropäische Brautechnische Analysenkommission (MEBAK) methods (MEBAK, 2012). A beer analyser system (DMA 4500, Alcolyzer) was used to analyse the EtOH concentration (Anton-Paar GmbH, Graz, Austria). A gas chromatograph coupled with a flame ionisation detector (GC–FID) was used to quantify esters and higher alcohols, according to MEBAK 2.21.6 guidelines in duplicates. Methyl hexanoate and 1-butanol were used as internal standards. Subsequently, EtOH (purity 99.5% abv) was added to the NAB sample to adjust the ethanol level to 5% abv. After determining the peak areas with the software Agilent ChemStation (Agilent Technologies, Waldbronn, Germany), calibration curves were used to calculate the aroma components’ concentrations. The aroma substances acetaldehyde, propanol-1, 2-methylpropanol, 3-methylbutanol and 2-methylbutanol (higher alcohols), as well as the esters ethyl acetate, isobutyl acetate, ethyl butanoate, 3-methylbutyl acetate, 2-methylbutyl acetate and ethyl hexanoate, were thus quantified.

### 2.4. Sensory Evaluation

The sensory evaluation of the NABs was conducted with a panel consisting of 13 members of the chair of brewing and beverage technology, trained once a week. All panellists were experienced in the sensory evaluation of beer and beer-based mixed beverages according to the DLG quality test guidelines [21]. The tasters’ task was to evaluate the intensities of specific attributes that characterise the aroma profile of NABs. These attributes were rated on a five-point, linear interval scale from 0 (not detectable) to 4 (very intense). The said attributes were selected from a ‘NAB term pool’ beforehand in a comprehensive panel training [22]. The panellists received 50 mL NAB samples identified by a random numerical code. The drinking temperature was 12 °C. The NAB samples were poured without foam. The aroma profiles of the tasted NABs are illustrated in a radar chart comprising the values for mean and standard deviation for the purpose of comparison.

### 2.5. Statistics

OriginPro 2020, version 9.7.0.188 (OriginLab Corporation, Northampton, MA, USA) was used to conduct statistical analyses and create the relevant figures. Tasting sessions were conducted with computer-aided sensory analysis tool FIZZ, version 2.60.00.1512 (Biosystèmes, Couternon, France). The mass balance diagrams were created using e!sankey^®^pro Version 3.2.2.558 (ifu Hamburg GmbH, Hamburg, Germany).

## 3. Results and Discussion

The parameters T_CON_ and $\dot{V}$_REFLUX_ were varied to determine their influence on the AW composition. The EtOH concentration of the material flows (beer OB, NAB, CON and AW) and the concentrations of the fermentation by-products in the AW were analysed. Balance sheets for EtOH and the fermentation by-products of the dealcoholisation process were created to clarify which flows carry the valuable aroma components. Based on these data, the degree of recovery of individual aroma components during the beer dealcoholisation process can be determined. To check the aroma enhancement and flavour optimisation hypothesis, a 0.0% abv NAB sample was blended with beer OB and AW independently reaching a value for the EtOH content of 0.5% abv.

### 3.1. Variation of the T_CON_ Value

The parameter T_CON_ influences the shift of valuable aroma substances in the aroma recovery. In fact, the value of T_CON_ determines whether aroma substances are discharged with EtOH into the CON or if they can be transferred to the aroma recovery. Indeed, condensation of the aroma substances in the aroma recovery system renders them available for re-aromatisation of the 0.0% abv NABs. If, however, EtOH and flavourings are mostly liquefied in the condenser, hardly any aroma components reach the aroma recovery system. In this case, the dealcoholisation equipment is not operating optimally.

T_CON_ was made to vary from 10 °C to 25 °C, while the temperature of the aroma recovery system remained constant at 8 °C. The EtOH concentrations in the beer OB sample and the NAB, CON and AW are listed in Table 1.

**Figure 2:**
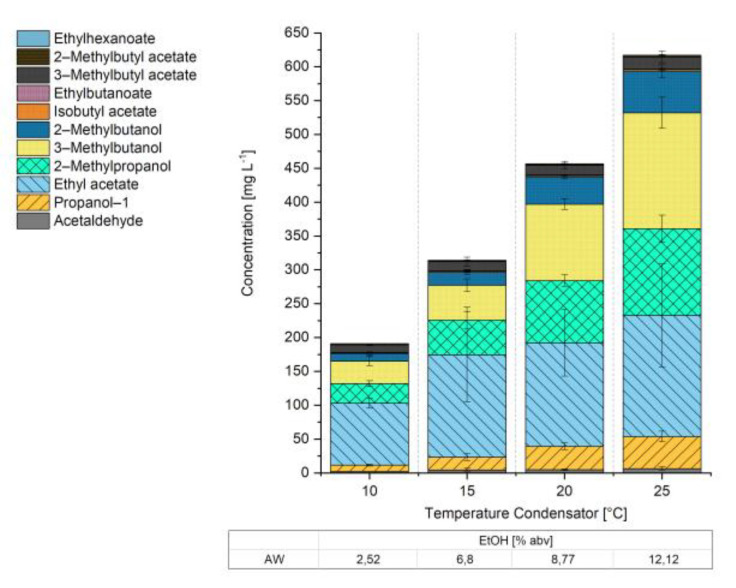
Individual and overall concentrations of the fermentation by-products present in the aromawater (AW) as a function of the condenser temperature. In the table at the bottom of the figure the corresponding ethanol (EtOH) concentration values of the AW are reported.

The data in this table indicate that most of the EtOH end up in the CON. With constant reflux at 20 L h^−1^, the maximum EtOH concentration in the CON (38.74% abv) was obtained at a T_CON_-value of 15 °C. At values for the T_CON_ above 15 °C, complete dealcoholisation of the beer was no longer possible. The data in Figure 2 provide information on how the concentrations of esters and higher alcohols are affected by the value of T_CON_.

A positive correlation was observed to exist between the T_CON_ value and the overall concentration of the aroma substances present in AW (r = 0.87, *p* < 0.001). In other words, an increase in the temperature of the condenser was observed to be associated with an increase in the total concentration of aroma substances in the AW. However, if the temperature of the condenser’s coolant (e.g., glycol) exceeded 15 °C, the low-boiling-point substances (EtOH) and high-boiling fraction (NAB) could not be separated sufficiently. In fact, at T_CON_ = 20 °C and at T_CON_ = 25 °C, the EtOH concentration in the NABs was >0.5% abv. Moreover, EtOH concentration in the CON decreased from 38.74% abv at T_CON_ = 15 °C to 22.50% abv at T_CON_ = 25 °C; by contrast, the EtOH concentration increased in the AW from 6.80% abv at T_CON_ = 15 °C to 12.12% abv at T_CON_ = 25 °C. As the T_CON_ value increased from 15 °C to 25 °C, a higher proportion of EtOH was no longer liquefied in the condenser but in the subsequent aroma recovery column. Compared to aroma recovery lab scale systems using pervaporation during beer [23] and wine [24,25] dealcoholisation, the enrichment of EtOH and aroma compounds differs. The residual level of EtOH in the thermal industrial-scale recovery unit used in this study is significantly lower (6.80% abv at the optimal operation conditions of T_CON_ = 15 °C) than the value for pervaporation process (28.67% abv) [23]. Therefore, the system used in the present study is more suitable for AW re-dilution into the NAB. Although implementation of aroma recovery via pervaporation makes it possible to achieve significantly higher levels of AW enrichment for higher alcohols and especially esters, the high EtOH concentration of the pervaporation-AW (28.67% abv) limits its re-use for NAB aromatisation. In industry scale systems like the DeAlcoTec^®^, the degree of concentration depends on the volume flow of the degassed water ($\dot{V}$_AroRec_). In this study, $\dot{V}$_AroRec_ was kept constant at 60 L h^−1^ to compensate the concentration during dealcoholisation [11].

In summary, the hypothesis that the concentrations of the constituent aroma compounds in the AW can be tuned by adjusting the T_CON_ value was confirmed. Importantly, the applied T_CON_ must not be at such a high level as to prevent the goal of obtaining a NAB product characterised by an EtOH concentration below 0.5% abv.

### 3.2. Variation of the $\dot{V}$_REFLUX_ Value

The $\dot{V}$_REFLUX_ value of the dealcoholisation process was made to vary from 0 to 60 L h^−1^. T_EVAP_ was kept constant at 36 °C, and the $\dot{V}$_AroRec_ value was 60 L h^−1^. The aim of these experiments was to investigate the dependence of the EtOH enrichment in the CON on the EtOH concentration in the AW. The T_CON_ was kept constant at 10 °C to ensure the dealcoholisation of the NAB (<0.05% abv).

The data listed in Table 2 indicate that the EtOH concentration in the various fractions increased in association with increments in the reflux rate. However, the ratio between the alcohol concentration in the AW and in the CON remains constant at ~10%, regardless of the chosen reflux rate. The values for the EtOH concentration in the NAB are not listed in Table 2 because they were in the 0.02–0.05% abv range. Therefore, the goal of complete dealcoholisation was achieved.

**Figure 3:**
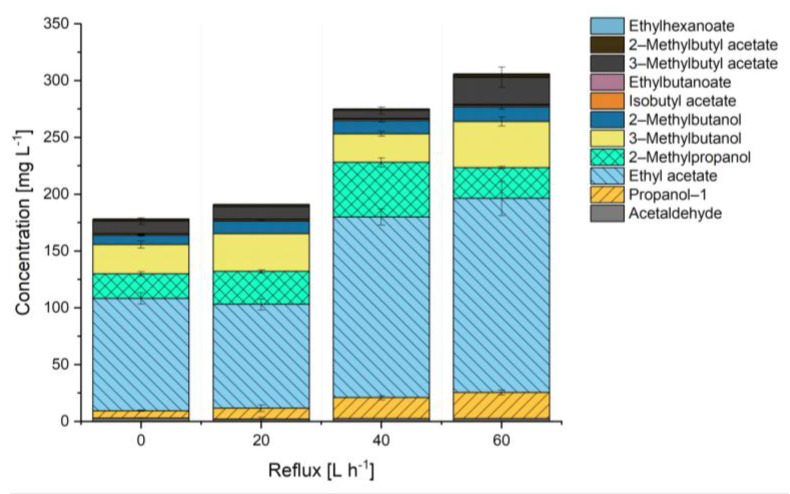
Individual and overall concentrations of the fermentation by-products present in the aromawater as a function of the value of the reflux rate (0–60 L h^−1^).

The concentrations of the individual aroma substances in the AW determined for different values of the $\dot{V}$_REFLUX_ are reported in Figure 3.

The aroma compounds’ concentration increased significantly as the value $\dot{V}$_REFLUX_ increased from 0 to 60 L h^−1^, and a positive correlation was observed to exist between the aroma concentrations and $\dot{V}$_REFLUX_; this correlation could be fitted by a second-order polynomial regression (R^2^ = 0.93). Specifically, the concentration of propanol increased from 6.4 mg L^−1^ at $\dot{V}$_REFLUX_ = 0 to 9.57 mg L^−1^ at $\dot{V}$_REFLUX_ = 20 L h^−1^, 18.50 mg L^−1^ at $\dot{V}$_REFLUX_ = 40 L h^−1^ and 22.90 mg L^−1^ at $\dot{V}$_REFLUX_ = 60 L h^−1^. The concentration of the valuable, banana-like ester 3-methylbutyl acetate (3-MBA), which is type-specific for top-fermented beers, increased from 11.0 mg L^−1^ at $\dot{V}$_REFLUX_ = 0 L h^−1^ to 24.1 mg L^−1^ at $\dot{V}$_REFLUX_ = 60 L h^−1^.

For comparison, in alcoholic beers, the concentration of 3-MBA is known to range from 0.5 to 8 mg L^−1^ [26]. In the beers OB used in the present study, the average 3-MBA concentration was 4.34 ± 0.26 mg L^−1^. During aroma recovery, esters like 3-MBA are enriched in the AW by a multiple of their concentration in the starting product. Notably, the AW enrichment factor was observed to depend on the chosen $\dot{V}$_REFLUX_ value; thus, for $\dot{V}$_REFLUX_ = 0 L h^−1^, the enrichment factor was 1.5, whereas for $\dot{V}$_REFLUX_ = 60 L h^−1^, the enrichment factor was 5. However, a thermal aroma recovery unit like the one used in this study cannot achieve a selective cut of EtOH and aroma substances. The presence of a residual amount of EtOH in the AW cannot be avoided and its level depends on the chosen T_CON_ and $\dot{V}$_REFLUX_ values.

Since the EtOH level is lower in the AW than in CON, the former can subsequently be added to the process for flavour enhancement. As different legal limits exist for EtOH in NABs (e.g., up to 1.2% abv in France), this is relevant as a natural flavouring for beverages in line with ‘clean labelling‘ [20].

### 3.3. Mass Balances for Valuable Ingredients during Thermal Dealcoholisation

**Figure 4:**
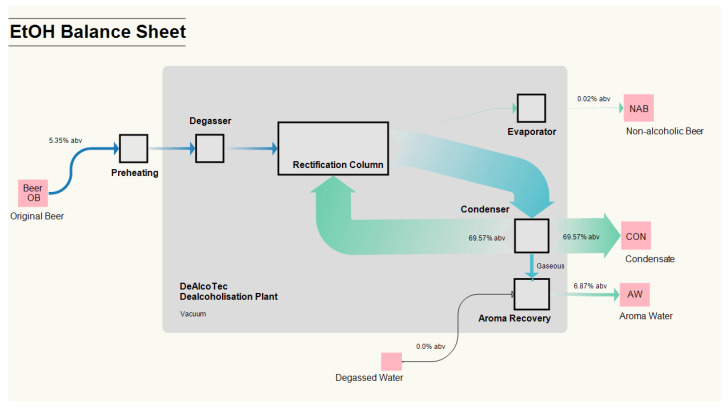
Balance sheet illustrating the distribution of ethanol in the DeAlcoTec^®^ thermal dealcoholisation system; the dealcoholisation experiments were conducted at an evaporation temperature of 36 °C, a condenser temperature of 10 °C and a reflux rate of 60 L h^−1^. The flow rate of degassed water was 60 L h^−1^. The pink squares represent the initial (beer OB) and final products (non-alcoholic beer NAB, condensate CON and aromawater AW) of the dealcoholisation process. The black squares/rectangles represent the single unit operations in the system. The isobaric area under vacuum is assumed to be the greyed system area.

In contrast to the preceding section, which focused on the concentration of aroma substances, this section focuses on the enrichment and depletion of selected substance groups during the dealcoholisation process. Due to the different unit operations (e.g., dealcoholisation, condensation and aroma recovery), the dealcoholisation process (EtOH, individual, higher alcohols and esters from a cumulative perspective) is subsequently shown as Sankey diagrams for overview purposes, see Figure 4.

In the dealcoholisation process, the CON is a by-product that comprises most of the high-boiling component EtOH. Using the parameter settings described in the caption of Figure 4, the separation and complete dealcoholisation of beer OB (NAB: 0.02% abv) was ensured. The AW obtained in the described experimental conditions is characterised by an EtOH level of 6.87% abv. NAB and AW containing valuable aroma substances for later re-use are the value products of the process. Most of the esters and higher alcohols are evaporated in the rectification column, passing to the vaporous part. Even if the total concentrations of esters and higher alcohols are higher in the CON than in the other fractions, the CON cannot be used for further processing in NAB production due to its high EtOH level. In fact, the aroma compounds present in the CON are discharged from the beverage production process. Nevertheless, further downstream treatment should be assessed for each brewery depending on CON’s quantity and purity. From the perspective of the re-use of AW in NAB production, the concentrations and the ratio of beer aroma substances in the AW and the enrichment of aroma compounds elucidating a positive, beer-typical are decisive.

**Figure 5:**
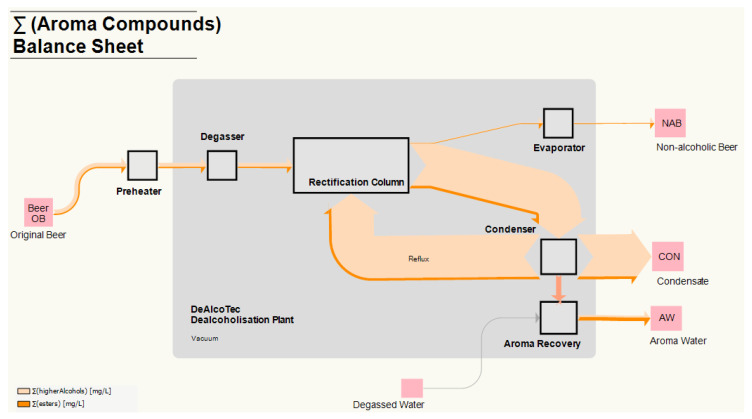
Balance sheet and distribution of the sum of esters (i.e., ethyl acetate, isobutyl acetate, ethylbutanoate, 3-methyl butyl acetate, 2-methylbutyl acetate and ethylhexanoate) and higher alcohols (e.g., propanol, 2-methylpropanol, 3-methylbutanol and 2-methylbutanol) in the DeAlcoTec^®^ thermal dealcoholisation system. The dealcoholisation experiment was conducted at an evaporation temperature of 36 °C, a condenser temperature of 10 °C and a reflux rate of 60 L h^−1^. The volume flow rate for degassed water was 60 L h^−1^. The pink squares represent the initial (beer OB) and final products (non-alcoholic beer NAB, condensate CON and aromawater AW) of the dealcoholisation process. The black squares/rectangles depict the single unit operations in the system.

As can be seen from Figure 5, the ratio of esters and higher alcohols differs between CON and AW. To evaluate the predominant enrichment of aroma substances, the concentrations of representative substances in CON and AW were correlated and compared with the c_REL_ of EtOH (Table 3). The percentage c_REL_ provides information on how much of the relevant substance remains in the CON for the chosen parameter setting and which proportion of it is transferred to the AW. The c_REL_ of 3-methylbutanol (3-MB), employed as a representative of higher alcohols, was calculated. In fact, 3-MB is considered a typical fermentation by-product in fermented beverages, such as wine or beer. Notably, as a pure substance 3-MB has a malty, vinous and pungent flavour [27]. However, the fruity, banana-flavoured 3-MBA, which is one of the desirable key aroma compounds of top-fermented beers, was chosen as a representative of esters [28,29].

Omitting the reflux rate, 13.05% of the CON concentration of 3-MB was detected in the AW. If a reflux rate of 60 L h^−1^ was applied, the percentage share was observed to be reduced to 3.54%. Overall, evidence suggests that as the reflux rate increases, higher alcohols are separated from the CON. The situation differs for 3-MBA. At the maximum reflux value of 60 L h^−1^, 67.13% of the CON concentration of 3-MBA was detected in the AW.

Associating the aroma component’s concentrations of CON and AW calculating c_REL_, evidence suggests that the aliphatic alcohols (e.g., propanol, 2-methylpropanol, 3-MB and 2-methylbutanol) accumulate primarily in the CON. The aroma recovery’s selectivity is on fermentation by-product esters (e.g., ethyl acetate and 3-MBA).

### 3.4. Use Case for Aroma Enhancement of NABs

**Figure 6:**
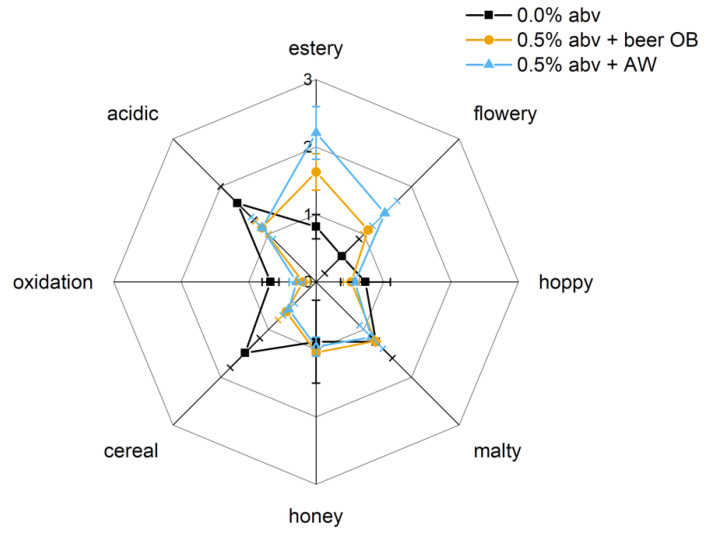
Radar diagram reflecting the sensory evaluation of a non-alcoholic beer (NAB) produced with the DeAlcoTec^®^ thermal dealcoholisation system and of two blends with original beer (beer OB) and aromawater (AW); the dealcoholisation experiment was conducted at an evaporation temperature of 36 °C, a condenser temperature of 10 °C and a reflux rate of 60 L h^−1^; in the radar diagram, the intensities are rated as follows: 1 = low, 2 = medium, 3 = strong $\left(\bar{x}\pm \sigma \right)$.

The question as to whether breweries will apply aroma recovery rests on the evaluation of whether the addition of the AW will lead to an aroma improvement for the NABs, and if the aroma will be comparable or rather more intense than with a blend with beer OB. To answer this question, sensory evaluation was performed on a 0.0% abv NAB, a blend with beer OB up to 0.5% abv and a blend with AW to 0.5% abv on aroma attribute intensities, typical of NABs (Figure 6).

A weak estery aroma and a medium cereal-like, acidic flavour characterised the 0.0% abv NAB sample. Blending this sample with beer OB led to an EtOH level of 0.5% abv. With the re-dilution of original beer (beer OB), the intensity of the attribute ‘estery’ increases. This result is also achieved by blending the 0.0% abv NAB sample with AW to an EtOH level of 0.5% abv. Flavours perceived as unpleasant, such as ‘cereal’ and an acidic taste, were masked by the addition of beer OB’s and AW’s fermentation by-products. The intensities for ‘estery’ and ‘flowery’ attributes were higher in the 0.5% abv + AW-blend than in the blend with beer OB.

The results of the conducted experimentsto evaluate the samples’ sensory attributes indicate that the intensification of an estery and flowery flavour and an improvement of the overall impression of 0.5% abv NABs is achievable by re-using the AW fraction. Moreover, a significant difference regarding non-volatile matrix attributes was not detectable.

## 4. Conclusions

Rectification systems for the thermal dealcoholisation of beer afford the possibility to recover valuable aroma substances via a downstream column. However, knowledge regarding how to influence the recovery fluid composition was lacking in terms of the type of aroma substances present and their concentrations by the choice of the parameters of the dealcoholisation process. If the aroma recovery procedure delivers an AW that does not meet the desired requirements regarding type and concentration of aroma compounds, as well as requirements for blending with the NAB such as microbiological status and lowest concentrations of oxygen, it is usually discarded. A further point of criticism is that the AW is not free from EtOH.

The aim of this study was to determine whether it is possible to influence the type and concentration of beer aroma components in the AW as part of the aroma recovery process.

The parameters T_CON_ and $\dot{V}$_REFLUX_ were varied during thermal dealcoholisation processes conducted using the DeAlcoTec^®^ system. The obtained results indicate that the concentration of aroma compounds can be increased by raising the T_CON_ value from 10 °C to 25 °C. However, if the T_CON_ value exceeds 15 °C, the complete dealcoholisation of the sample is not achievable anymore. On the other hand, raising the $\dot{V}$_REFLUX_ value leads to an increase in EtOH concentration in the CON and AW, although the percentage share c_REL_ of EtOH remains constant, at 10%. The concentration of valuable esters and higher alcohols in the AW can be made to increase significantly by raising $\dot{V}$_REFLUX_.

Determining the c_REL_ values for the aroma substances 3-MB, as a representative for higher alcohols and 3-MBA, as a representative for esters provides evidence that increasing the reflux rate leads to a predominant enrichment of higher alcohols in the CON and valuable esters in the AW. The concentrations and yields of aroma substances can differ depending on the initial product type of variety.

The preparation of NAB blends aiming an improved product aroma is the most obvious field of application of the results of the present study. The estery aroma, which is typical for top-fermented wheat beers, could be intensified by adding AW to the NAB. Low volumes of beer-typical aroma substances are already used to mask off-flavours of 0.0% abv NABs. This effect is more pronounced when the NAB samples are blended with AW than being blended with beer OB. Further downstream processing of the NAB samples for obtaining versatile aroma concentrates is feasible; for instance, as a supplement for hard seltzers.

In summary, the results of this study indicate that aroma recovery only displays its advantages when the temperature levels in the condenser and the aroma recovery column are matched. Thus far, the aroma substances obtained as a by-product of the thermal dealcoholisation process have not been considered for their use in NAB production from an economic point of view. The recovery of these aroma substances represents an attractive alternative for the production of high value-added products, which can be labelled as natural aromas if recovered appropriately.

## Figures and Tables

**Table 1:** Ethanol concentration $\left(\bar{x}\pm \sigma \right)$ in the original product (beer OB) and in the products obtained after the thermal dealcoholisation process: non-alcoholic beer (NAB), condensate (CON) and the fluid of the aroma recovery ‘aromawater’ (AW). The process was performed at different values for the condenser temperature (T_CON_).

	T_CON_ [°C]	10	15	20	25
		$\bar{x}\pm \sigma$	$\bar{x}\pm \sigma$	$\bar{x}\pm \sigma$	$\bar{x}\pm \sigma$
**Beer OB**	[% abv]	5.26 ± 0.01	5.28 ± 0.03	5.28 ± 0.03	5.28 ± 0.03
**NAB**	[% abv]	0.06 ± 0.05	0.17 ± 0.07	1.17 ± 0.33	1.25 ± 0.19
**CON**	[% abv]	28.23 ± 0.39	38.74 ± 2.19	35.84 ± 3.19	22.50 ± 0.28
**AW**	[% abv]	2.52 ± 0.39	6.80 ± 0.19	8.77 ± 0.47	12.12 ± 2.15

**Table 2:** Ethanol (EtOH) concentration $\left(\bar{x}\pm \sigma \right)$ in the condensate (CON) and aromawater (AW) at different values for the reflux rate; c_REL_ (EtOH) represents the percentage ratio between the EtOH concentration in the AW to its level in the CON.

**Reflux Rate**	[L h^−1^]	0	20	40	60
**Temperature**	[°C]	10	10	10	10
**EtOH (CON)**	[% abv]	22.80 ± 0.53	27.57 ± 2.87	42.40 ± 1.12	69.57 ± 7.79
**EtOH (AW)**	[% abv]	2.28 ± 0.17	2.19 ± 0.29	4.02 ± 0.14	6.87 ± 0.62
**c_REL_ (EtOH)**	[%]	9.61	8.45	9.54	9.50

**Table 3:** Values for the c_REL_ of ethanol (EtOH) and of the aroma compounds 3-methylbutanol (3-MB), used as a representative of higher alcohols, and 3-methylbutyl acetate (3-MBA), used as a representative of esters, given as a function of the chosen reflux rate. The temperature of the condenser remained constant at 10 °C. c_REL_ represents the percentage ratio between the concentration of the particular compound in the aromawater and the one in the condensate.

**Reflux Rate**	[L h^−1^]	0	20	40	60
**c_REL_ (EtOH)**	[%]	9.61	8.45	9.54	9.50
**c_REL_ (3-MB)**	[%]	13.05	12.18	3.75	3.54
**c_REL_ (3-MBA)**	[%]	57.89	50.78	50.00	67.13
